# Glyphosate Herbicide Induces Changes in the Growth Pattern and Somatic Indices of Crossbred Red Tilapia (*O. niloticus × O. mossambicus*)

**DOI:** 10.3390/ani11051209

**Published:** 2021-04-22

**Authors:** Umar Abubakar Muhammad, Nur Adeela Yasid, Hassan Mohd Daud, Mohd Yunus Shukor

**Affiliations:** 1 Department of Biochemistry, Faculty of Biotechnology and Biomolecular Sciences, University Putra Malaysia (UPM), Seri Kembangan 43400, Malaysia; abubakar.umar1980@gmail.com (U.A.M.); adeela@upm.edu.my (N.A.Y.); 2 Department of Biological Sciences, Faculty of Science, Gombe State University, P.M.B. 027, Gombe State 760214, Nigeria; 3 Department of Veterinary Clinical Studies, Faculty of Veterinary Medicine, University Putra Malaysia (UPM), Seri Kembangan 43400, Malaysia; hassanmd@upm.edu.my

**Keywords:** glyphosate, technical grade, ecotoxicity, red tilapia, toxicity parameters

## Abstract

**Simple Summary:**

In this study, a chronic, seven-week study of the effect of technical grade glyphosate on the toxicity parameters of crossbred red tilapia (*O. niloticus × O. mossambicus*) was carried out. The results show that the bodyweight index was the most sensitive toxicity parameter wherein a reduction in body weight was observed at 25 mg/L of glyphosate. Negative correlations between the glyphosate concentration and toxicity parameters such as specific growth rate (SGR), hepato-somatic index (HIS), and gonado-somatic index (GSI) were observed. The fish condition factor and feed conversion ratio were found to be unaffected at the highest glyphosate concentration tested (150 mg/L).

**Abstract:**

The development of glyphosate-resistant genetically modified organisms (GMO) has increased the use of herbicide glyphosate by several magnitudes in recent years. It is now the most commonly used pesticide globally that affects aquatic habitats, especially fish. This study aims to add new knowledge on the effect of technical grade glyphosate on several toxicity parameters and to identify the most effective parameter in predicting technical grade glyphosate chronic toxicity (seven weeks) to fish, especially Malaysia’s heavily farmed red tilapia. The results show that a relatively high concentration of technical grade glyphosate is needed to induce significant changes in all tested parameters. However, the results also indicate that the bodyweight index is the most sensitive toxicity parameter in that a reduction in body weight was observed at 25 mg/L of glyphosate. Negative correlations between the glyphosate concentration and toxicity parameters such as specific growth rate (SGR), hepato-somatic index (HIS), and gonado-somatic index (GSI) were observed. The fish condition factor and feed conversion ratio were found not to be affected at the highest glyphosate concentration tested (150 mg/L). To conclude, crossbred red tilapia (*O. niloticus × O. mossambicus*) is one potential species for evaluating the toxic effects of technical grade glyphosate on fish.

## 1. Introduction

Water contamination as a result of agrochemical applications of pesticides is considered one of the major challenges to the conservation of aquatic ecosystems [1,2]. Globally, pesticidal pollution, especially run-offs from agricultural areas, is a major concern due to the acute and chronic toxicities for aquatic organisms [3,4]. Chronic and acute exposures to pesticides in large quantities can have adverse effects on the fish physiology, biochemistry, and population stability, as well as the overall health of the ecosystem [5,6,7]. The continuous movement of glyphosate and components of its formulations into aquatic habitats can influence the aquatic inhabitants in various ways, such as occasioning fish death when exposed to a high dose or being deposited in a manner that results in chronic damage to aquatic inhabitants [8,9,10,11].

Glyphosate formulation can be 10, 40, or even 100 times more toxic than pure or technical grade glyphosate. For example, in *Daphnia magna*, the 96 h LC_50_ of pure glyphosate is 962 mg/L, while for the formulation Roundup^®^, the LC_50_ drops to 25.5 mg/L [12]. However, of the components of the commercial formulation, glyphosate is the most soluble whilst the surfactants in the formulation such as polyethoxylated tallow amine (POEA) have poor solubility. Hence, soil water runoffs would contain pure glyphosate more than other components such as surfactants in many glyphosate formulations, as the latter’s washout from the soil is assumed to be minimal due to their low water solubility and strong soil sorption capacity [13]. The environmentally relevant concentration of glyphosate is about 0.4 mg/L [14]. Despite this, studies based on actual water runoffs from fields freshly applied with glyphosate formulation have demonstrated glyphosate concentrations as high as 5.2 mg/L [15], whilst simulated studies using sand as a matrix showed water runoffs containing as high as 17 mg/L glyphosate [16]. Hence, several studies have utilized high concentrations of the glyphosate formulation Roundup^®^, e.g., 20 mg/L for the fish *Leporinus obtusidens* (piava) [14], 100 mg/L for the brine shrimp (*Artemia salina*) nauplii [17], and between 100 and 500 mg/L for the embryo of zebrafish [18]. For pure glyphosate, concentrations such as 26.3 mg glyphosate per kg soil for compost worms (*Eisenia fetida*) [19] and 40 mg/L for the juveniles of crayfish *Cherax quadricarinatus* [20] have been utilized. 

The measurement of toxicity parameters has often involved a multi-marker approach such as body mass indices, food conversion ratio, specific growth rate, hepato-somatic index (HSI), gonado-somatic index (GSI) [21,22], and hematological parameters [23,24]. The condition factor is one of the body mass indices that explain the relationship between the length and weight of the fish [9]. This factor is regarded as a somatic biomarker indicating the level of well-being and overall health conditions of the fish in their aquatic environment [9,25]. It can also serve as a vital tool for monitoring changes in the growth pattern of fish and the condition of an aquatic environment [26], as well as being generally applied to evaluate the effects of environmental stressors on fish. Stress can reduce the condition factor of the fish which can be translated into a decrease in fat or deposited glycogen in the fish liver [27]. The gonado-somatic index (GSI) is the percentage of gonad weight and fish weight ratio [3]. GSI is the change in gonad weight to the percentage mass of the fish [28]. The hepato-somatic index (HSI) is the percentage of liver weight and fish mass ratio, which is widely applied in fisheries as a biomarker of liver energy storage and a useful parameter for monitoring the effects of toxicants on fish [29,30]. In fish and other animals, vitellogenesis (i.e., the process of vitellogenin synthesis) is the link between GSI and HSI [31,32]. Routine contact of the fish with sub-lethal dosages of pesticides alters the growth performance, survival rate, hepato-somatic index, and immunity of the fish [33].

Red tilapia is a hybrid mutant of *Oreochromis niloticus* and *Oreochromis mossambicus*. It is a great cultivable species with an immense global market. This is attributed to its attractive beauty, good taste, ease of cultivation, strong capacity to respond to environmental stresses, high disease tolerance, omnivorous eating patterns, short generation time, and quick growth. It is the dominant farmed species (85%) in Malaysia due to the above attributes [34,35]. The present study aims to investigate changes in the growth and somatic indices of crossbred red tilapia (*O. niloticus × O. mossambicus*) after exposure to a technical grade glyphosate herbicide. As the toxicity of technical grade glyphosate is far less than that of glyphosate formulation, this study was conducted using concentrations of up to 150 mg/L so as to ascertain the concentration level at which technical grade glyphosate can have an impact on the toxicological parameters of this fish. To the best of our knowledge, this is the first such study using technical grade glyphosate on this organism.

## 2. Materials and Methods

### 2.1. Animal Ethics Approval and Consent

Before the commencement of this study, approval was granted by the Universiti Putra Malaysia Institutional Animal Care and Use Committee with the approval number UPM/IACUC/AUP-R033/2017.

### 2.2. Determination of Water Quality Parameters

The physicochemical parameters of the aquaria water used for the experiment such as temperature, dissolved oxygen, and hydrogen ion concentration (pH) were determined periodically [36].

### 2.3. Experimental Design and Glyphosate Treatment

Healthy fish of both sexes (about four months of age) were procured from a registered fish farmer in Selangor, Malaysia and conveyed to the laboratory under safe conditions. The fish were acclimatized for 10 days in non-chlorinated aquarium water. The average length of the fish ranged between 18 and 23 cm and the average weight was between 155 and 166 g. The fish were fed with Dinding’s commercial feed pellets (2.5 mm) (Dindings Soya & Multifeeds Sdn Berhad) at 4% body weight two times daily. The weight of the daily feed given was determined using an electronic weighing balance (Model GF-3000, Muser Apac Sdn Bhd, Kuala Lumpur, Malaysia) and recorded in grams. Following feeding, the remaining pellets were removed from the aquarium (36.5 cm × 25 cm × 26 cm glass aquarium) using a scoop net and dried, and the weights were determined in order to ascertain the actual weight of the feed consumed by the fish in each aquarium every day. Feeding was stopped 24 h prior to the commencement of the experiment [37]. Fish (ten fish per dosage, five male and female per dosage) were subjected to five glyphosate dosages (0, 25, 50, 100, and 150 mg/L) for 49 days. Technical grade glyphosate was purchased from Fluka BioChemika (Sigma, St. Louis, MO, USA). The water and glyphosate content was changed every 4 days to conserve a healthy environment and constant glyphosate concentration in the water. The photoperiod was 12L:12D.

### 2.4. Fish Growth Analysis

The fish growth pattern was determined morphometrically, which included measurements of length and weight, condition factor, food conversion ratio, and specific growth rate. Five fish were randomly selected from each concentration and the control group for the purpose of the experiment [38]. The length and weight of the fish were measured periodically while the fish was anaesthetized. Fish were anaesthetized using clove oil (0.1 mL/L) [39]. Ethanol was used to dissolve the clove oil because it is not soluble in water. The initial (K1) and final (K2) fish condition factor were calculated according to the method of [40];

Fish Condition factor (K) = (W × 100)/L^3^ (1)

where

W = weight of the fish in grams;
L = length of the fish in centimeters.

The food conversion ratio (FCR) and specific growth rate (SGR) were determined using the following equations:

Food Conversion Ratio (FCR) = Dry food fed (g)/Weight gained (g)(2)

Specific Growth Rate (SGR) = Log final body weight − Log initial body Weight × 100 Time (in days)(3)

### 2.5. Determination of Organ-Somatic Indices of Fish

At the end of glyphosate toxicity testing, the weight of the fish from control and treated fish was determined for somatic indices analysis. Fish were anaesthetized by tricaine methane-sulfonate (MS-222; 30 mg/L), sedated, and slaughtered. After dissection, the liver and gonads moisture was dried using a blotting paper. The weights of the liver and the gonads were measured using an electronic weighing balance (Model: GF-3000) and recorded in grams. The hepato-somatic index was determined as the ratio of liver weight to the weight of the fish expressed in percentage [41], whereas the gonado-somatic index was determined as the ratio of gonad mass to the mass of the fish expressed in percentage [42]. The organ-somatic indices of fish were calculated as follows:

Hepato-somatic index (HSI, %) = 100 × liver weight (g)/body weight (g)(4)

Gonado-somatic index (GSI, %) = 100 × Gonad weight (g)/body weight (g) (5)

### 2.6. Statistical Analysis

Analysis of variance (ANOVA) using SAS version 4.9 (SAS Institute, Cary, NC, USA) was used to compare the results between the control and fish exposed to different glyphosate concentrations and post hoc was done using Tukey’s multiple comparison test. The data met the assumption of homogeneity of variance. Linear correlation between variables was also carried out using the Pearson correlation coefficient.

## 3. Results

### 3.1. Body Mass Indices

The temperature, pH, and dissolved oxygen levels throughout the experiment were normal at 19.5 ± 1.0 °C, 7.22 ± 0.28, and 8 ± 0.82 mg/L, respectively. The effects of chronic exposure to glyphosate on the length and weight of red tilapia were studied for 49 days (7 weeks). There was a general trend of reduction in length and weight, as the fish were exposed to higher glyphosate concentrations with a reduction in length. This was observed as early as week 3 at the highest glyphosate concentration tested (150 mg/L) (Table 1). It appears that the effect of glyphosate was more pronounced on fish weight as a significant reduction in weight occurred as early as one week into the experimental period at the highest glyphosate concentration tested (150 mg/L). At the lowest glyphosate concentration, no reduction in length was observed at all during the experimental period whilst a reduction in weight was seen at week 3 (Table 2) at the lowest concentration of glyphosate (25 mg/L). At the end of the experiment, no mortality or gross behavioral changes were observed aside from fish treated with the highest glyphosate concentration (150 mg/L) showing sluggish behavior and being less active than the controls, with a few of the treated fish found resting at the bottom of the aquaria from time to time.

### 3.2. Fish Condition Factor

**Figure 1:**
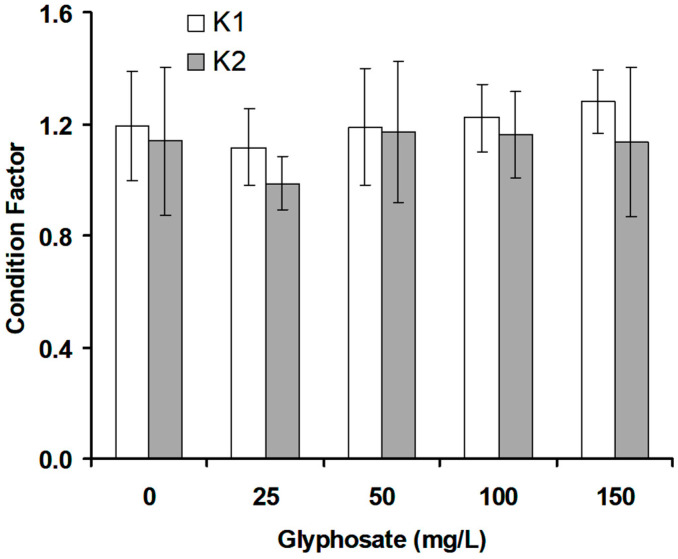
Changes in condition factor of crossbred red tilapia (*O. niloticus × O. mossambicus*) due to glyphosate exposure. Error bars represent the mean ± SD, n = 3.

The effect of glyphosate on the condition factor was investigated (Figure 1). There were no changes (*p* > 0.05) in the initial (K1) and final (K2) conditional factors of the fish at any of the glyphosate concentrations tested.

**Figure 2:**
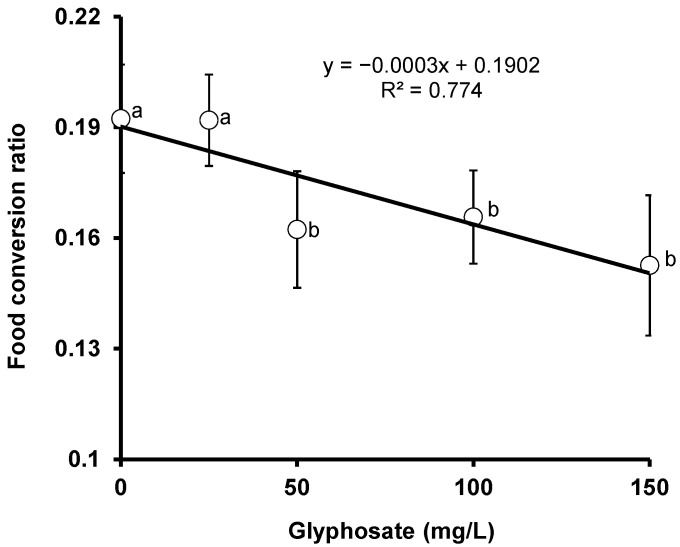
Changes in the food conversion ratio of crossbred red tilapia (*O. niloticus × O. mossambicus*) due to glyphosate exposure. Error bars represent the mean ± SD, n = 3. Means with the same letter are not significantly different (*p* > 0.05).

Figure 2 shows the results of the effects of glyphosate on the food conversion ratio of red tilapia. A significant negative correlation (*r* = −0.879, *p* = 0.0491) was observed between the food conversion ratio and glyphosate concentration. The p value was close to 0.05, indicating nearly no significant difference despite the relatively high value of the correlation coefficient value. The figure revealed an insignificant (*p* < 0.05) difference between the control and the fish subjected to a lower glyphosate dose of 25 mg/L, but with a remarkable (*p* < 0.05) difference between the control and fish subjected to higher concentrations of 50 to 150 mg/L, indicating the dangers posed by technical grade glyphosate on red tilapia at high concentrations. 

### 3.3. Specific Growth Rate (SGR)

**Figure 3:**
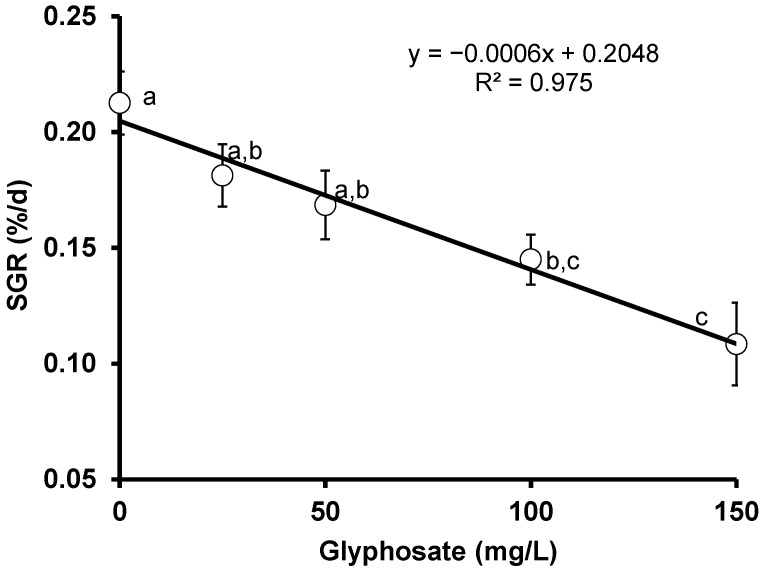
Changes in the specific growth rate of crossbred red tilapia (*O. niloticus × O. mossambicus*) due to glyphosate exposure. Error bars represent the mean ± SD, n = 3. Means with the same letter are not significantly different (*p* > 0.05).

The specific growth rate in fish is considered a tool mostly used in aquaculture for the estimation of fish production over a certain period of time. The result of the consequences of glyphosate herbicide exposure for the specific growth rate of red tilapia shows a slight but insignificant (*p* > 0.05) difference between the untreated and fish treated with 25 and 50 mg/L of glyphosate herbicide. A significant (*p* < 0.05) difference emerged between the control and the fish exposed to 100 and 150 mg/L, indicating the harm of increasing the glyphosate dose on the specific growth rate of red tilapia. A significant negative correlation (*r* = −0.987, *p* = 0.0017) was observed between the specific growth rate (SGR) and glyphosate concentration (Figure 3). 

### 3.4. Hepato-Somatic Index (HSI)

**Figure 4:**
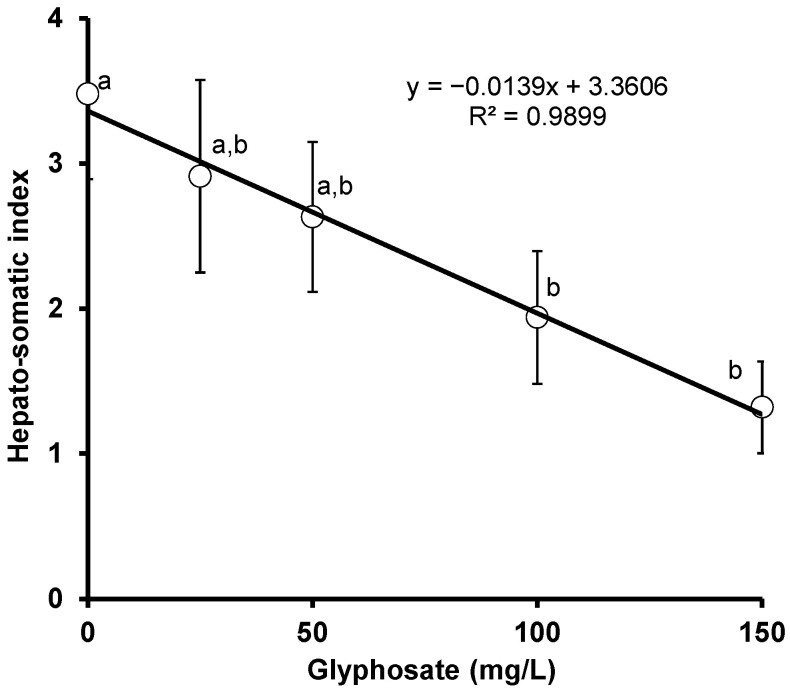
Changes in the HSI of crossbred red tilapia (*O. niloticus × O. mossambicus*) due to glyphosate exposure. Error bars represent the mean ± SD, n = 3. Means with the same letter are not significantly different (*p* > 0.05).

The effect of glyphosate on the hepato-somatic index of red tilapia was determined. A largely significant (*p* < 0.05) change in HSI was observed in the present study. A significant negative correlation (*r* = −0.995, *p* = 0.0004) was observed between HSI and glyphosate concentration (Figure 4). 

### 3.5. Gonado-Somatic Index (GSI)

**Figure 5:**
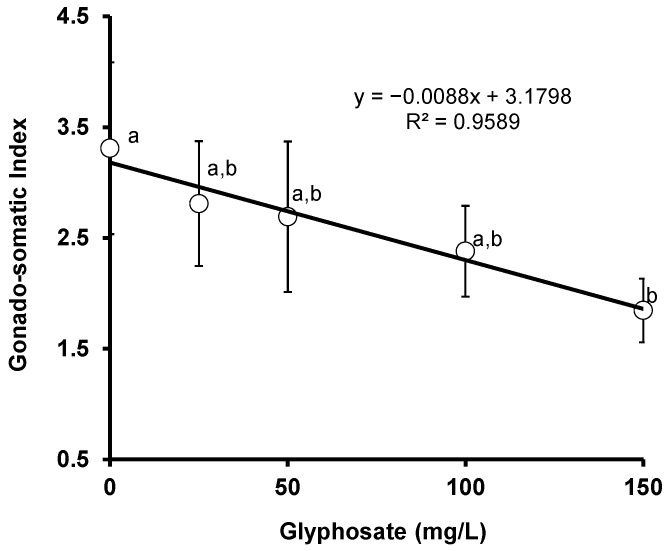
Changes in the GSI of crossbred red tilapia (*O. niloticus × O. mossambicus*) due to glyphosate exposure. Error bars represent the mean ± SD, n = 3. Means with the same letter are not significantly different (*p* > 0.05).

The results of the effects of glyphosate exposure on the gonado-somatic index of red tilapia are displayed. A significant (*p* < 0.05) change was detected in the GSI between the control and exposed red tilapia during the exposure periods. A significant negative correlation (*r* = −0.979, *p* = 0.0036) was observed between GSI and glyphosate concentration (Figure 5). 

## 4. Discussion

Throughout this study, no mortality or distressing behavioral changes were observed. This means that a comparison of observed toxic parameters with the endpoints’ behavioral observation could not be made. In a similar study wherein *Oreochromis niloticus* was exposed chronically to Roundup at between 5 and 15 mg/L, no observable mortality or significant behavioral changes were observed [43]. Since Roundup is generally more toxic to fish than the technical grade glyphosate utilized in this study, this result is within our expectations. There are only very few studies available on the effect of glyphosate (either pure or in the form of formulation) on body mass indices such as length or weight. In one study, *Clarias gariepinus* exposed to 5 mg/L of the glyphosate formulation for 28 days underwent a 7% reduction in weight [44] as was observed in this study but at a higher concentration of glyphosate. In another study on atrazine, a 60 day exposure to fathead minnow (*Pimephales promelas*) showed a 5% reduction in length [45]. On the other hand, crucian carp (*Carassius auratus*) exposed to alachlor showed no differences in the level of body length and body weight for all groups after 60 days of exposure [46]. After a 75 day exposure to bisphenol-S, one of the emerging flame retardant pollutants [47,48], the body length and weight of zebrafish were significantly decreased [49]. In a 20 day exposure to another flame retardant BDE-47, a reduction in the body mass of zebrafish was observed but not body length [50]. The results in this study and those from the literature suggest that body mass is a better biomarker for a toxicant than body length. As the body mass was found to decline at the lowest concentration of glyphosate tested (25 mg/L), further chronic studies are needed at much lower concentrations down to the environmentally relevant concentrations, which should be combined with an increase in exposure period in order to assess any possible negative effect that glyphosate might have on the body mass of this fish that can be translated as a decline in aquaculture yield.

A literature search would show only limited information on correlational studies between the effect of glyphosate and body mass indices or toxic parameters. Correlational studies in toxicity research based on the Pearson correlation coefficient in an attempt to draw a link between the concentration of toxicants and toxic parameters have been carried out on several occasions. These include the effect of the organophosphate pesticide fenitrothion on the behavioral alterations of European seabass *Dicentrarchus labrax* [51] or the effect of total toxic compounds in ponds on the reproductive capacity of the common toad *Bufo bufo* [52]. In some of the correlation studies, a nonlinear correlation is implied as seen in a study on the effect of chlorpyrifos on the specific growth rate of the American lobster (*Homarus americanus*) [53] or the effect of nitrite on the total feed intake and specific growth rate of *Clarias gariepinus* [54]. In this study, we attempt to correlate the effect of technical grade glyphosate to several toxicity parameters. The first is the condition factor (CF), which is commonly known to be a physiological index of fish growth, reflecting a classical indicator of health and fitness according to fish biologists for fish population screening [55]. CF is used to offer accurate details on fish species residing in a sub-optimal setting, wherein fish with CF >1 are regarded as showing isometric growth and being in excellent health [56,57,58]. CF is also accompanied by the organosomatic indices (organ weight ratio to body weight), where both indices can differ naturally with food supply, sexual maturation status, and life history period [59]. As CF is considered a valid reflection of the health status of an individual by showing the correlation between the physiological and environmental condition, a considerably lower CF dose upon exposure to stressors may be observed [57,60]. Lower CF values upon exposure to pesticides have been reported in rainbow trout exposed to a mixture of ten pesticides [61], mummichog (*Fundulus heteroclitus*) larvae exposed to atrazine under salinity stress conditions [62], and red mullets from a polluted area of the Spanish Mediterranean coast [63]. The results show a correlation between a decline in condition factor and an increase in glyphosate concentration. In another experiment, no significant change was observed for the condition factor between the control group and fish treated with dietary copper [64]. No changes to the CF values were also reported in *Clarias gariepinus* exposed to p,p′-DDT at an environmentally relevant concentration [65] or *Clarias gariepinus* exposed to cypermethrin [41]. However, *C. gariepinus* is a hardy fish [66]. The above findings may be explained by two primary factors. Firstly, CF is a measure of symptoms at a higher degree of biological sophistication, which can only happen when animals are under the highest stressed conditions. Secondly, CF is primarily affected by naturally fluctuating factors such as age and breeding activity, and as such these factors could have masked the effect of CF changes [67]. Stress due to exposure to a toxicant such as glyphosate can reduce the condition factor of the fish, which can result from an increase in the rate of body metabolism, reduction in energy intake, and/or a decrease in body fat or stored glycogen in the liver [27]. 

The negative correlation coefficient for the food conversion ratio observed in this study is barely significant, such that an insignificant difference to the control is the conservative approach to be concluded. In other similar studies, no significant change in the food conversion ratio was reported between the control and the exposure of *Oncorhynchus mykiss* to copper over a period of 3 months [68] and *Oreochromis niloticus* to dietary copper for 42 days [64]. On the other hand, copper supplemented in the feed of blunt snout bream (*Megalobrama amblycephala*) resulted in a reduction of the feed conversion ratio [69]. Similarly, *C. gariepinus* exposed to lead for 6 weeks showed a dramatic decline in the food conversion ratio [70]. As far as the effect of glyphosate exposure to the food or feed conversion ratio in fish is concerned, very little to no studies have been reported.

There is a dearth of information on the effect of glyphosate (either technical grade or formulation) on the specific growth rate of fish or SGR in chronic studies on red tilapia or even fish in general. One study has demonstrated a linear decline of SGR in *C. gariepinus* exposed to varying concentrations of glyphosate formulation at up to 5 mg/L for 28 days [44]. A similar trend was reported due to the exposure of crayfish (*Cherax quadricarinatus*) to glyphosate and polyoxyethylamine [71]. A similar reduction of SGR upon exposure to other pesticides such as dimethoate and malathion was reported for Nile tilapia (*O. niloticus* L.) [72]. In another study, after exposure to the technical grade (94% a.i.) and commercial formulations (20% EC) of chlorpyrifos, *O. niloticus* showed a reduction in SGR after a 90 day exposure period [73]. The specific growth rate of fish is a tool mostly used in aquaculture for the estimation of fish production over a certain period of time. The reduction in SGR can be related to the increase in stress due to exposure to glyphosate as mentioned above. 

The decline in the HSI was also reported in sub-adult *O. niloticus* subjected to a glyphosate herbicide [74]. A significant decrease in the hepato-somatic index of *Heteropneustes fossils* was also reported after contact with sub-lethal dosages of arsenic and copper [75]. Significant decreases in the GSI and HIS indices were observed when crucian carp (*Carassius auratus*) were exposed to alachlor for 60 days [46]. After 75 days of exposure, there was a decrease in the GSI among zebrafish exposed to bisphenol-S [49]. GSI and HSI could have sensitive toxic effect or could be indicator of pollution or toxicant exposure since they are easy parameters to measure. On the other hand, there were no significant changes in the hepato-somatic index of *C. gariepinus* subjected to atrazine [76]. In another study, *C. gariepinus* exposed to lead acetate showed no significant difference in the HSI after 6 weeks of exposure [70], which may be related to this species being a hardy fish. The downward trend observed in the GSI indicates that glyphosate herbicide exerted a significant negative effect on the gonads as well as the reproductive ability of the red tilapia. Similarly, a significant change was reported in the gonado-somatic index of sub-adult *O. niloticus* due to glyphosate herbicide exposure [76]. Exposure to aquatic toxicants is reported to cause a significant change in the gonado-somatic indices of fish species [75,77]. However, no significant change was reported in the GSI of *C. gariepinus* subjected to atrazine, *Cocos nucifera* water, or *Phyllanthus muelarianus* extract [76]. Both the HIS and GSI were found to significantly decline only at a relatively high concentration of glyphosate (100 mg/L), which is far higher than the environmentally relevant concentration of glyphosate, indicating that these indices might not have played a significant role.

## 5. Conclusions

There is a dearth of information on the effects of pure and technical grade glyphosate, or even glyphosate formulation, on some of the most popular fish ecotoxicity parameters, such as body mass index, condition factors, feed or food conversion factor, GSI, and HIS. This study aims to add new knowledge on the effect of technical grade glyphosate on these ecotoxicity parameters and to ascertain which of these parameters is the most effective in predicting technical grade glyphosate toxicity to fish, especially Malaysia’s heavily farmed red tilapia. The results show that relatively high concentrations of technical grade glyphosate are needed to induce significant changes in all parameters tested, which could be expected for pure and technical grade glyphosates. The results also indicated that, of the body mass indices comprising length and weight, the bodyweight index was the most sensitive toxicity parameter wherein a significant reduction in body weight was found at 25 mg/L of glyphosate whilst condition and feed conversion factors were not sensitive parameters. Of the other parameters tested, such as the specific growth rate (SGR), HIS, and GSI, the most sensitive was SGR. Current works include a study on the effect of lower concentrations of technical grade glyphosate (less than 25 mg/L) down to the environmentally relevant concentration on the body mass of this fish within a longer period of study especially at full maturity before harvesting (30 to 34 weeks) so as to determine whether prolonged exposure can affect toxicity parameters and aquaculture yield. To conclude, crossbred red tilapia (*O. niloticus × O. mossambicus*) has proven to be a potential species for evaluating the chronic effects of technical grade glyphosate toxicity to fish.

## Figures and Tables

**Table 1:** Chronic effect of glyphosate on crossbred red tilapia (*O. niloticus × O. mossambicus*) length (cm) at various growth periods. Values are mean ± standard deviation (n = 5). Values having the same letter in the same row are not significantly different (*p* > 0.05).

	0 mg/L	25 mg/L	50 mg/L	100 mg/L	150 mg/L
Week 0	21.20±0.84 ^a^	21.20 ± 0.84 ^a^	21.40 ± 0.55 ^a^	21.20 ± 0.84 ^a^	21.00 ± 1.00 ^a^
Week 1	23.80 ± 0.84 ^a^	23.40 ± 2.07 ^a^	23.00 ± 1.58 ^a^	22.00 ± 1.41 ^a^	21.40 ± 0.55 ^a^
Week 2	25.60 ± 1.14 ^a^	24.00 ± 2.24 ^a,b^	23.80 ± 1.64 ^a,b^	23.00 ± 1.58 ^a,b^	22.40 ± 0.55 ^b^
Week 3	27.20 ± 0.84 ^a^	25.20 ± 1.30 ^a,b^	24.40 ± 2.07 ^b^	23.80 ± 1.10 ^b^	23.00 ± 0.71 ^b^
Week 4	27.60 ± 0.55 ^a^	26.20 ± 0.84 ^a,b^	25.20 ± 1.64 ^b,c^	25.00 ± 0.71 ^b,c^	24.20 ± 1.10 ^c^
Week 5	27.80 ± 1.10 ^a^	27.00 ± 1.00 ^a,b^	25.80 ± 1.48 ^a,b,c^	25.00 ± 0.71 ^b,c^	23.80 ± 0.84 ^c^
Week 6	28.20 ± 0.84 ^a^	27.80 ± 0.45 ^a^	25.40 ± 1.14 ^b^	26.00 ± 0.71 ^b,c^	24.40 ± 0.55 ^c^
Week 7	29.00 ± 0.71 ^a^	28.60 ± 0.55 ^a^	26.00 ± 0.71 ^b^	26.00 ± 0.71 ^b^	24.80 ± 1.10 ^b^

**Table 2:** Chronic effect of glyphosate on the weight (g) of crossbred red tilapia (*O. niloticus × O. mossambicus*) at various growth periods. Values are mean ± standard deviation (n = 5). Values having the same letter in the same row are not significantly different (*p* > 0.05).

	0 mg/L	25 mg/L	50 mg/L	100 mg/L	150 mg/L
Week 0	159.00 ± 4.85 ^a^	159.20 ± 3.83 ^a^	155.20 ± 8.70 ^a^	157.80 ± 4.15 ^a^	153.80 ± 2.59 ^a^
Week 1	167.80 ± 3.19 ^a^	166.60 ± 3.91 ^a,b^	162.80 ± 2.39 ^a,b^	161.00 ± 3.39 ^b,c^	155.60 ± 1.82 ^c^
Week 2	171.20 ± 2.77 ^a^	170.60 ± 4.83 ^a^	167.20 ± 4.32 ^a,b^	163.20 ± 3.11 ^b,c^	157.80 ± 1.92 ^c^
Week 3	183.80 ± 2.59 ^a^	173.00 ± 1.87 ^b^	172.60 ± 5.77 ^b^	167.20 ± 4.09 ^b,c^	161.00 ± 3.16 ^c^
Week 4	189.60 ± 5.94 ^a^	178.00 ± 3.39 ^b^	175.00 ± 3.54 ^b,c^	170.40 ± 3.36 ^c,d^	163.60 ± 1.67 ^d^
Week 5	194.00 ± 4.00 ^a^	182.60 ± 5.77 ^b^	175.80 ± 3.49 ^b,c^	174.40 ± 3.78 ^c^	169.20 ± 3.42 ^c^
Week 6	196.60 ± 4.16 ^a^	192.60 ± 2.97 ^b^	181.00 ± 4.74 ^c^	177.60 ± 2.88 ^c,d^	172.80 ± 2.28 ^d^
Week 7	199.25 ± 6.02 ^a^	191.00 ± 2.16 ^b^	181.60 ± 2.07 ^c^	181.60 ± 2.07 ^c^	175.40 ± 2.79 ^d^

## Data Availability

The data presented in this study are openly available in FigShare.com, doi:10.6084/m9.figshare.13664795 with the link https://figshare.com/search?q=10.6084%2Fm9.figshare.13664795 (accessed on 16 April 2021).
